# Bone morphogenetic protein 2 inhibits the proliferation and growth of human colorectal cancer cells

**DOI:** 10.3892/or.2014.3308

**Published:** 2014-07-03

**Authors:** YUNYUAN ZHANG, XIAN CHEN, MIN QIAO, BING-QIANG ZHANG, NING WANG, ZHONGLIN ZHANG, ZHAN LIAO, LIYI ZENG, YOULIN DENG, FANG DENG, JUNHUI ZHANG, LIANGJUN YIN, WEI LIU, QIAN ZHANG, ZHENGJIAN YAN, JIXING YE, ZHONGLIANG WANG, LAN ZHOU, HUE H. LUU, REX C. HAYDON, TONG-CHUAN HE, HONGYU ZHANG

**Affiliations:** 1 Ministry of Education Key Laboratory of Clinical Diagnostic Medicine and the Affiliated Hospitals of Chongqing Medical University, Chongqing 400016, P.R. China; 2 Molecular Oncology Laboratory, Department of Orthopaedic Surgery, The University of Chicago Medical Center, Chicago, IL 60637, USA; 3 Department of Laboratory Medicine, The Affiliated Hospitals of Qingdao University, Qingdao 266003, P.R. China; 4 Departments of Cell Biology and Oncology, Affiliated Southwest Hospital, Third Military Medical University, Chongqing 400038, P.R. China; 5 Department of Surgery, Affiliated Zhongnan Hospital of Wuhan University, Wuhan 430071, P.R. China; 6 Departments of Orthopaedic Surgery and General Surgery, The Affiliated Xiang-Ya Hospital of Central South University, Changsha 410008, P.R. China; 7 School of Bioengineering, Chongqing University, Chongqing 400044, P.R. China

**Keywords:** colorectal cancer, BMP2, BMP signaling, tumorigenesis, proliferation, intestinal epithelial cells

## Abstract

Colorectal cancer (CRC) is one of the most deadly cancers worldwide. Significant progress has been made in understanding the molecular pathogenesis of CRC, which has led to successful early diagnosis, surgical intervention and combination chemotherapy. However, limited therapeutic options are available for metastatic and/or drug-resistant CRC. While the aberrantly activated Wnt/β-catenin pathway plays a critical initiating role in CRC development, disruption of the bone morphogenetic protein (BMP) pathway causes juvenile polyposis syndrome, suggesting that BMP signaling may play a role in CRC development. However, conflicting results have been reported concerning the possible roles of BMP signaling in sporadic colon cancer. Here, we investigated the effect of BMP2 on the proliferation, migration, invasiveness and tumor growth capability of human CRC cells. Using an adenovirus vector that overexpresses BMP2 and the piggyBac transposon-mediated stable BMP2 overexpression CRC line, we found that exogenous BMP2 effectively inhibited HCT116 cell proliferation and colony formation. BMP2 was shown to suppress colon cancer cell migration and invasiveness. Under a low serum culture condition, forced expression of BMP2 induced a significantly increased level of apoptosis in HCT116 cells. Using a xenograft tumor model, we found that forced expression of BMP2 in HCT116 cells suppressed tumor growth, accompanied by decreased cell proliferation activity. Taken together, our results strongly suggest that BMP2 plays an important inhibitory role in governing the proliferation and aggressive features of human CRC cells.

## Introduction

Colorectal cancer (CRC) causes an average of 50,000 deaths per year in the US and has emerged as the second leading cause of cancer-related mortality in the US and worldwide (1,2). While early diagnosis and surgical intervention, along with combination chemotherapy, has led to improved outcomes, few effective strategies have emerged with which to treat colon cancer once first-line approaches have been exhausted (1,2). The initiation and progression of CRC development are characterized by the accumulation of growing numbers of genetic and epigenetic changes (3,4), while the aberrant activation of the Wnt pathway, either by inactivation of tumor-suppressor adenomatous polyposis coli (APC) or oncogenic activation of β-catenin, has been demonstrated as the essential initial step of tumorigenesis (3,5). Nonetheless, other alternative pathways have been implicated in CRC development. One involves the formation of serrated adenomas that are associated with mutations in BRAF (6). Another alternative pathway involves the formation of a hamartoma as a precursor lesion, which is in this last rare pathway to CRC that mutations in the bone morphogenetic protein (BMP) pathway were identified (7).

BMPs belong to the transforming growth factor β (TGFβ) superfamily (8). BMPs bind to a heterodimeric complex of transmembrane serine threonine kinase receptors type 1 and 2, triggering the phosphorylation and activation of the type 1 receptor by the type 2 receptor kinase. The activated type 1 receptor phosphorylates a receptor-associated SMAD which subsequently complexes with SMAD4 and translocates to the nucleus to regulate gene transcription (9). The importance of BMP signaling in colon cancer development has been highlighted by the identification of mutations in the BMP pathway in colorectal carcinogenesis (7). *SMAD4* was identified as being frequently deleted in CRC, although the biological significance of this genetic change has always been attributed to loss of TGFβ signaling rather than BMP signaling (10). Mutations in BMP receptor 1A (*BMPR1A*) were found in patients with juvenile polyposis (JP), a rare autosomal dominant hamartomatous polyposis syndrome with an increased risk for the development of CRC (11). Mutations in *SMAD4* and *BMPR1A* account for approximately half of all cases of JP (12–14). Moreover, forced expression of the BMP antagonist noggin in the mouse intestine results in the formation of intestinal hamartomatous polyps (15).

However, conflicting results have been reported concerning the possible roles of BMPs in sporadic colon cancer. For example, several BMPs were found to be growth suppressive and may have their promoters methylated in colon cancer, compatible with a tumor-suppressor role for BMPs in CRC (16–18). However, the expression of BMP4 and BMP7 was found to increase with progression through the adenoma-carcinoma sequence and to correlate with a worse prognosis (19,20). A more recent report showed that BMP signaling promotes the growth of primary human colon cancer *in vivo* (21). Therefore, the biological effects of BMPs on colon cancer development and progression remain to be fully elucidated.

In the present study, we investigated the effect of BMP2 on the proliferation, migration, invasiveness and tumor growth capabilities of human colon cancer cells. To achieve high levels of exogenous BMP2 expression, we constructed an adenovirus vector that overexpresses BMP2 and also generated the piggyBac transposon-mediated stable BMP2 overexpression cell line using the commonly used human colon cancer line HCT116. We found that exogenous BMP2 effectively inhibited HCT116 cell proliferation and colony formation. BMP2 was shown to suppress colon cancer cell migration and invasiveness as assessed by cell wound healing assay and Boyden chamber Transwell assay. Under a low serum condition, forced expression of BMP2 induced a significantly higher percentage of apoptosis in HCT116 cells than that in the controls. Using a xenograft tumor model, we found that forced expression of BMP2 in HCT116 cells suppressed tumor growth, accompanied by decreased proliferative activity. Thus, our results strongly suggest that BMP2 may play an important inhibitory role in controlling the proliferation and aggressive features of colon cancer cells.

## Materials and methods

### Cell culture and chemicals

Human colon cancer cell lines HCT116 and HEK-293 were obtained from the American Type Culture Collection (ATCC; Manassas, VA, USA). The cells were maintained in complete DMEM containing 10% fetal bovine serum (FBS; Hyclone, Logan, UT), 100 units of penicillin and 100 μg of streptomycin at 37°C in 5% CO_2_ as previously reported (22–27). Unless otherwise indicated, all chemicals were purchased from Sigma-Aldrich (St. Louis, MO, USA) or Thermo Fisher (Pittsburgh, PA, USA).

### Recombinant adenoviral vectors expressing BMP2 or GFP

Recombinant adenoviruses were generated using AdEasy technology (28–32). Briefly, the coding regions of human BMP2 and green fluorescent protein (GFP) were PCR amplified and cloned into adenoviral shuttle vectors, which were subsequently used to generate recombinant adenoviruses in HEK-293 cells as previously described (29,32). The resultant recombinant adenoviruses were designated as AdGFP and AdBMP2, respectively. The amplified adenoviruses were titrated and stored at −80°C.

### Establishment of BMP2/FLuc and FLuc expression stable cell lines

In order to construct BMP2 and/or firefly luciferase (FLuc) stable expression cell lines, the coding regions of human BMP2 and/or FLuc were PCR amplified and subcloned into a homemade *piggyBac* vector pMPB5, resulting in pMPB-BMP2/FLuc and pMPB-FLuc, respectively. The PCR amplified sequences were verified by DNA sequencing. To construct stable cell lines, exponentially growing HCT116 cells were co-transfected with pMPB-BMP2/FLuc or pMPB-FLuc and the Super *piggyBac* transposase expression vector (System Biosciences, Mountain View, CA, USA) using Lipofectamine transfection reagents by following the manufacturer’s instructions (Life Technologies, Grand Island, NY, USA). At 24 h after transfection, stable clones were selected in the presence of blasticidin S (10 μg/ml) for 5 days. The resultant stable cell lines were designated as HCT116-BMP2/FLuc and HCT116-FLuc, respectively. The stable cell lines were verified by RT-PCR for BMP2 expression and/or firefly luciferase activity assay.

### Colony formation assay

Exponentially growing HCT116 cells were seeded in 6-well plates at a low density (300 cells/well) and infected with AdGFP or AdBMP2 (MOI=20) for 2 weeks to form colonies. The medium was replaced every 3–4 days. The uninfected cells were also included as a control. The colonies were stained with crystal violet. Each assay condition was conducted in triplicate and repeated in at least three batches of independent experiments. The average colony number for each group was calculated and expressed as the colony formation rate (colony number/seeded cell number) × 100%.

### Cell proliferation (MTT) assay

In order to assess cell proliferation and viability, the MTT [3-(4,5-dimethylthiazol-2-yl)-2,5-diphenyltetrazolium bromide] assay was performed as previously described (33–39). Briefly, subconfluent HCT116 cells were infected with AdBMP2 or AdGFP (MOI=20) for 16 h and seeded in 96-well plates (1,000 cells/well). The plated cells were incubated in DMEM supplied with 1% FBS. At the indicated time points, the cells were incubated with 10 μl of the CellTiter 96^®^ Non-Radioactive Cell Proliferation Assay (MTT) reagent (Promega, Madison, WI, USA) at 37°C for 4 h, followed by addition of 100 μl DMSO to dissolve the formazan products for 10 min at room temperature with gentle agitation. The absorbance was measured at 492 nm using a microtiter plate reader. Each assay condition was carried out in five replicates. The overall experiments were repeated at least in three batches of independent experiments.

### Cell migration/wound healing assay

Subconfluent HCT116 cells were infected with AdGFP or AdBMP2 for 16 h and reseeded in 6-well plates at ~90% confluency. Upon cell attachment, scratches were made with pipette micro-tips. Floating cells were removed and the attached cells were maintained in DMEM supplemented with 1% FBS. The width of the scratched cell gaps were monitored and recorded at different time points. The scratch assay was carried out in triplicate and at least three scratch sites were monitored and recorded in each well. Percentage of the wound area closure was measured using ImageJ software.

### Boyden chamber invasion/migration assays

The Matrigel cell invasion assay was performed as previously described (33,34,40). Briefly, subconfluent HCT116 cells were infected with AdGFP or AdBMP2 for 24 h. Polycarbonate membranes with 8-μm pores were coated with Matrigel (BD Biosciences). The membranes were rehydrated, and 5×10^5^ of the transduced cells were placed onto each upper chamber of the Transwell unit. Medium with 10% FBS was used as a chemoattractant in the bottom chamber. The cells were allowed to invade at 37°C in 5% CO_2_ for 24 h. Cells were fixed in 10% formalin and washed with PBS. The cells were stained with hematoxylin and rinsed with water. Cells on the unmigrated side were gently wiped off with a wet cotton tip applicator, and the membrane was rinsed with water. The membranes containing the migrated cells were dried and mounted onto slides with Permount. The number of migrated cells per high power field (HPF) was determined by averaging 20 randomly counted HPFs. The assays were performed in triplicate and repeated in at least three batches of independent experiments.

### Apoptosis and flow cytometric analysis

Subconfluent HCT116-FLuc and HCT116-BMP2/FLuc cells were cultured in DMEM containing 1% FBS for 72 h. Both floating and attached cells were collected, stained with Annexin V-FITC and propidium iodide (PI) using the Annexin V-FITC apoptosis detection kit (BD Pharmingen™, BD Biosciences). The stained cells were subjected to FACS analysis using the BD™ LSR II flow cytometer and FlowJo software. Each assay was performed in triplicate and repeated at least three times.

### Xenograft tumor growth and xenogen whole body bioluminescence imaging

All animal experiments reported in this study were carried out in strict accordance with the recommendations established in the Guide for the Care and Use of Laboratory Animals of the National Institutes of Health. The protocol was approved by the Institutional Animal Care and Use Committee (IACUC). For subcutaneous xenograft tumor formation, 4–6 week old male athymic nude (nu/nu) mice were purchased from Harlan Sprague Dawley (Indianapolis, IN, USA). Exponentially growing HCT116-BMP2/FLuc and HCT116-FLuc cells were harvested and resuspended in PBS. Cells (2×10^6^ in 100 μl of PBS) were injected subcutaneously into the flanks of athymic mice (n=6/group). All animals were sacrificed 4 weeks after injection.

For weekly whole body bioluminescence imaging, the animals were anesthetized with isoflurane attached to a nose-cone mask within the Xenogen IVIS 200 imaging system. Mice were injected with D-Luciferin sodium salt (Gold BioTechnology, St. Louis, MO, USA) at 100 mg/kg in 0.1 ml sterile PBS. The pseudo-images were obtained by superimposing the emitted light over the grayscale images of the animal. The average signals in photons/sec/cm^2^/steradian were calculated. Quantitative analysis was carried out using the Xenogen’s Living Image software as previously described (22,35,38,41,42).

### Histologic evaluation and immunohistochemical staining

The retrieved tissues were fixed in 10% buffered formalin and embedded in paraffin. The 5-μm sections were subjected to H&E staining. Immunohistochemistry was carried out as previously described (22,23,30,31,40,43–46). For immunohistochemical staining, sections were deparaffinized, rehydrated, subjected to antigen retrieval and probed with a PNCA antibody (Santa Cruz Biotechnology), followed by incubation with biotin-secondary antibodies and streptavidin-HRP. PCNA protein was visualized by 3,3′-diaminobenzidine staining. Control IgG and minus-primary antibody staining were used as negative controls.

### Statistical analysis

All quantitative data were calculated and are expressed as means ± standard deviation. The differences between groups were analyzed using one-way ANOVA followed by the Student-Newman-Keuls test using GraphPad Prism software. A P<0.05 was considered to indicate a statistically significant result.

## Results and Discussion

### Exogenous BMP2 inhibits the proliferative activity of human colon cancer cells

**Figure 1:**
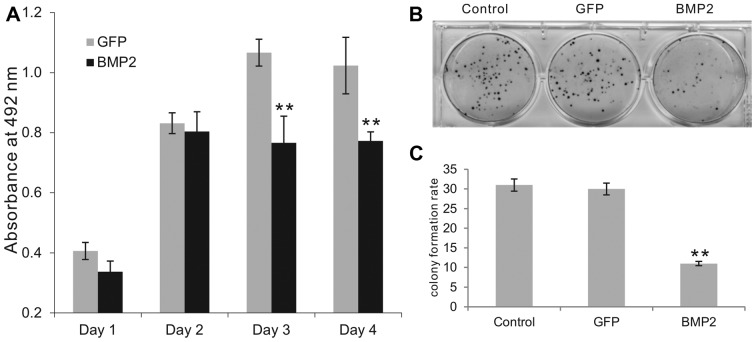
BMP2 inhibits the proliferation and colony formation capability of human colon cancer cells. (A) MTT assay. HCT116 cells were infected with AdGFP or AdBMP2 (MOI=20), seeded in 96-well plates and cultured in DMEM containing 1% FBS for 4 days. The cells were subjected to MTT assay by measuring the absorbance at 492 nm using a microplate reader. Each assay condition was carried out in triplicate. ^**^P<0.001 (BMP2 vs. GFP control). (B and C) Colony formation assay. Exponentially growing HCT116 cells were infected with AdGFP or AdBMP2 (MOI=20) and seeded at a low cell density for 2 weeks to form colonies. The uninfected cells were also included as a control. The colonies were stained with crystal violet. (B) Representative images are shown. (C) Average colony numbers for each group were calculated and are expressed as colony formation rate (colony number/seeded cell number) × 100%. Each assay condition was carried out in triplicate and repeated in at least three batches of independent experiments. ^**^P<0.001 (BMP2 vs. GFP control).

As the effects of BMP2 on colon cancer cells remain to be fully understood, we used an adenoviral vector overexpressing BMP2 and investigated its effects on the cell proliferation of colon cancer HCT116 cells. Using MTT assay, we found that AdBMP2-infected HCT116 cells exhibited lower proliferative activity at all tested time points when compared with that of the AdGFP-transduced cells, although only the differences on days 3 and 4 exhibited statistical significance (P<0.001) (Fig. 1A). When the AdBMP2, AdGFP or uninfected HCT116 cells were seeded at a very low density and allowed to form colonies, the BMP2-expressing HCT116 group formed significantly fewer colonies (Fig. 1B). Quantitatively, the BMP2-expressing HCT116 group formed approximately one third of the number of colonies when compared with the number in the GFP or uninfected control groups (Fig. 1C). These results suggest that BMP2 inhibits the proliferative activity of human colon cancer cells.

### BMP2 inhibits the cell migration capability and invasiveness of human colon cancer cells

**Figure 2:**
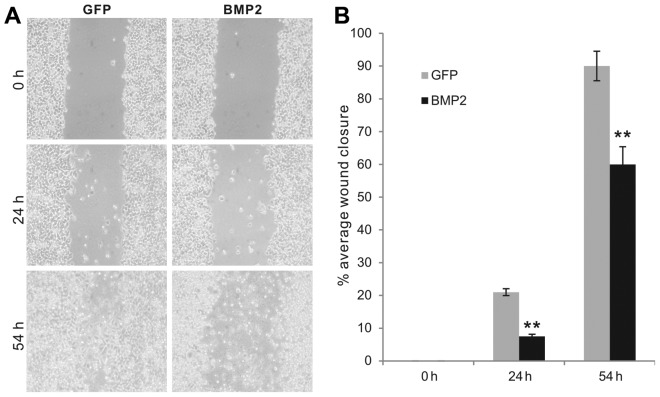
BMP2 inhibits the cell wound healing capability of human colon cancer cells. Subconfluent HCT116 cells were infected with AdGFP or AdBMP2 for 16 h and reseeded in 6-well plates at ~90% confluency. Upon cell attachment, scratches were made with pipette micro-tips. Floating cells were removed, and the cells were maintained in DMEM containing 1% FBS. The width of the scratched cell gaps were monitored and recorded at the indicated time points. The scratch assay was carried out in triplicate and at least three scratch sites were monitored and recorded in each well. (A) Representative images are shown. (B) Percentages of the wound area closure were measured using ImageJ software. Percentages of average wound closure for each group at different time points were graphed. ^**^P<0.001 (BMP2 vs. GFP).

We further examined whether BMP2 affects the migration capability and invasiveness of colon cancer cells. We performed the commonly used cell wound healing assay to assess the effect on cell migration. The AdBMP2-transduced HCT116 cells were shown to close the scratched gaps on monolayer culture at a much slower pace than that of the GFP control groups (Fig. 2A). The percentage of wound closure was significantly higher in the GFP-transduced control cells at all tested time points (P<0.001) (Fig. 2B). Thus, these results suggest that exogenous BMP2 expression may significantly inhibit the migratory capability of colon cancer cells.

**Figure 3:**
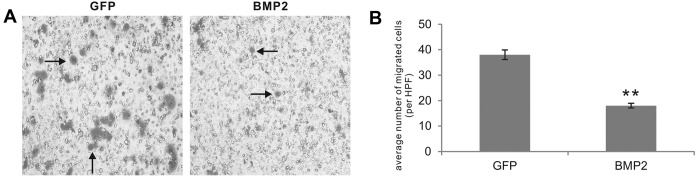
BMP2 inhibits the invasiveness and migratory ability of human colon cancer cells in Boyden chamber invasion/migration assays. Subconfluent HCT116 cells were infected with AdGFP or AdBMP2 for 24 h. The transduced cells were collected and seeded onto 8-μm pore Transwell polycarbonate membranes coated with a layer of Matrigel. The cells were allowed to migrate across the membrane using fetal bovine serum as a chemoattractant. Cells that have not migrated across were removed, and the migrated cells were formalin-fixed and stained with H&E. (A) The numbers of migrated cells were counted under a high power field (HPF, ×100) using a microscope. Each assay was carried out in triplicate. The cells migrated across the membrane are indicated by arrows. (B) At least 20 HPFs for each group were counted and the average numbers of the migrated cells were calculated and graphed. ^**^P<0.001 (BMP2 vs. GFP).

Using the Boyden Transwell extracellular matrix invasion assay, we analyzed the effect of BMP2 on the invasiveness of colon cancer cells. Consistent with our previous reports, GFP-treated HCT116 control cells were fairly aggressive and invaded the Matrigel-coated Transwell membrane with high efficiency, which was inhibited by exogenous BMP2 (Fig. 3A). Quantitatively, the BMP2-transduced HCT116 cells exhibited approximately <50% of the number of invaded cells in the GFP control group (P<0.001) (Fig. 3B), suggesting that BMP2 exerts an inhibitory effect on the invasiveness of colon cancer cells.

### BMP2 effectively induces apoptosis in human colon cancer cells

**Figure 4:**
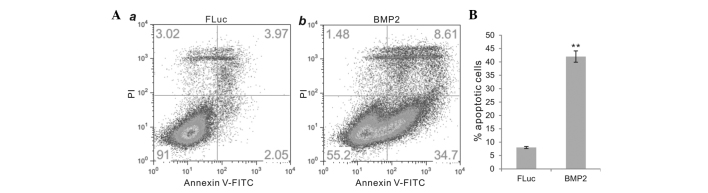
BMP2 effectively induces apoptosis in human colon cancer cells. (A) Subconfluent HCT116-FLuc (a) and HCT116-BMP2/FLuc (b) cells were cultured in DMEM containing 1% FBS for 72 h. The cells were collected, stained with Annexin V-FITC and propidium iodide (PI) and subjected to FACS analysis. Each assay was carried out in triplicate. (B) Percentages of apoptotic cells were calculated and graphed. ^**^P<0.001 (BMP2 vs. FLuc control).

We next investigated whether BMP2 induces apoptosis in colon cancer cells. We established a stable cell line HCT116-BMP2/FLuc that co-expressed human BMP2 and firefly luciferase (FLuc), while a control stable cell line HCT116-FLuc that only expresses FLuc was established in the same fashion. The exogenous expression of BMP2 was verified by RT-PCR analysis while the FLuc activity was determined using luciferase assay kits. We observed that HCT116-BMP2/FLuc cells grew normally in complete DMEM (with 10% FBS), compared with the parental HCT116 or HCT116-FLuc cells (data not shown). However, when the BMP2-expressing HCT116 cells were grown under no (0%) or low (1%) FBS condition, a significant increase in apoptosis was detected using the Annexin V labeling assay (Fig. 4A, panel a vs. b). When cultured in 1% FBS/DMEM for 72 h, the BMP2-expressing HCT116 cells underwent significant apoptosis (~42%), compared to ~8% in the control group (P<0.001) (Fig. 4B). Thus, these results suggest that BMP2 inhibits colon cancer cell proliferation at least in part through induction of apoptosis.

### BMP2 effectively inhibits the growth of xenograft tumors derived from human colon cancer

**Figure 5:**
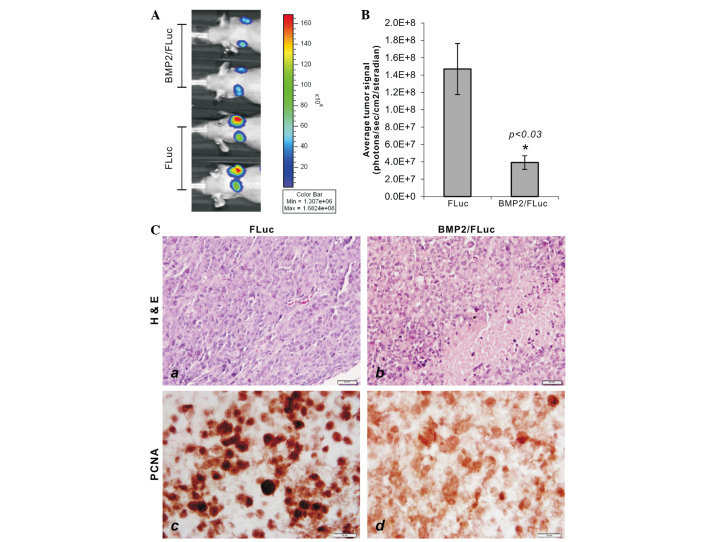
BMP2 inhibits tumor growth in a xenograft model of human colon cancer. (A and B) Xenograft tumor model of human colon cancer. Subconfluent HCT116-FLuc (FLuc) and HCT116-BMP2/FLuc (BMP2/FLuc) cells were collected, resuspended in PBS and subcutaneously injected into athymic nude mice (n=6; 2×10^6^ cells/injection). (A) Tumor growth was monitored weekly by whole body bioluminescence using the Xenogen IVIS 200 unit. (B) Quantitative data were obtained and analyzed (at week 4). (C) Histologic evaluation and immunohistochemical staining of the proliferation marker PCNA. The retrieved xenograft tumor samples were fixed, paraffin-embedded and sectioned for H&E staining (a and b, scale bar, 20 μm) and immunohistochemical staining with a PCNA antibody (c and d, scale bar, 10 μm). Control IgG and no primary antibody were used as controls (data not shown). Representative results are shown.

Although the above *in vitro* data strongly suggest that BMP2 exhibits an inhibitory effect on colon cancer cell proliferation, we aimed to verify whether the inhibitory effect could be extended to *in vivo* tumor models. We previously demonstrated that HCT116 cells can reproducibly form subcutaneous tumors in athymic nude mice (35,36). We used the FLuc-tagged stable lines, HCT116-FLuc and HCT116-BMP2/FLuc. Subconfluent HCT116-FLuc and HCT116-BMP2/FLuc (BMP2) cells were subcutaneously injected into athymic nude mice, and the tumor growth was monitored at weeks 2 and 4 using whole body xenogen bioluminescence. We found that the BMP2-expressing HCT116 group formed significantly smaller tumor masses at each time point (Fig. 5A). Quantitative analysis revealed that the tumor growth in the BMP2-expressing HCT116 tumors was ~26% when compared with the control group (P<0.03) (Fig. 5B). When the retrieved tumor samples were fixed, embedded and sectioned for H&E staining, the samples from the BMP2/FLuc group exhibited significant necrosis and low cell proliferation, compared with these parameters in the FLuc control group (Fig. 5C, panel a vs. b). The embedded samples were also sectioned and subjected to immunohistochemical staining with a PCNA antibody. We found that the tumor samples formed by HCT116 cells expressing BMP2 exhibited a significantly diminished staining of PCNA expression in the tumor cells (Fig. 5C, panel c vs. d), suggesting that the BMP2-expressing colon cancer cells have a decreased proliferative activity. Collectively, these *in vivo* results further confirm that BMP2 exhibits strong inhibitory effects on colon cancer cells, possibly through inhibiting their proliferation and migration and inducing apoptosis.

### BMP signaling may play an important role in modulating colorectal tumorigenesis

The findings from our *in vitro* and *in vivo* studies demonstrated that exogenous BMP2 inhibits cell proliferation, migration and invasion, induces apoptosis and suppresses *in vivo* xenograft tumor growth of human colon cancer cells. The importance of BMP signaling in colorectal tumorigenesis has been highlighted by the identification of frequent mutations of *SMAD4* in CRC (10) and mutations in BMP receptor 1A (*BMPR1A*) in patients with juvenile polyposis (JP) (12–14), which is associated with an increased risk for the development of CRC (11). Moreover, forced expression of the BMP antagonist noggin in mouse intestine was found to result in the formation of intestinal hamartomatous polyps (15).

Consistent with our findings are previous reports in which BMP2, BMP3 or BMP7 were shown to have growth-suppressive activities in colon cancer cells (16–18), although the expression of BMP4 and BMP7 was found to correlate with a worse prognosis (19,20). Notably, a more recent report showed that BMP signaling promotes the growth of primary human colon cancer *in vivo* and the investigators proposed that blockade of BMP signaling may have beneficial effects against at least a subset of advanced colon cancers (21). Nonetheless, studies have revealed that genetic variations in the BMP signaling pathway may be associated with the etiology, survival and/or prognosis of colon and rectal cancer (18,47–49).

Mechanistically, an early study suggested that BMP2 may act as a tumor suppressor promoting apoptosis in mature colonic epithelial cells (16), although it was suggested that BMP may also utilize SMAD4-independent pathways for growth suppression in colon cancers (18). Notably, it was reported that statins, acting as DNMT inhibitors can demethylate the BMP2 promoter, activate BMP signaling, induce differentiation of colon cancer stem cells and reduce their ‘stemness’ (50). Moreover, BMP-induced growth suppression may be mediated in part by p21^WAF1^, which is inhibited by RAS/ERK, as in colon cancer cells where BMP-SMAD signaling and growth suppression are facilitated by p21^WAF1^ but diminished by oncogenic K-RAS (51). It has been reported that suppression of the PI3 kinase/Akt pathway may be correlated with the development of BMP2 resistance and invasion in BMP2-induced epithelial-to-mesenchymal transformation (EMT) in colon cancer (52). It has been reported that the anti-mitogenic effect of proteasome inhibitors on colon cancer cells may require BMP signaling (53).

In summary, we investigated the effect of BMP2 on the proliferation, migration, invasiveness and tumor growth capabilities of human colon cancer cells. We found that exogenous BMP2 effectively inhibited HCT116 cell proliferation and colony formation. BMP2 also suppressed colon cancer cell migration and invasiveness. Forced expression of BMP2 induced significant apoptosis in HCT116 cells. Using an xenograft tumor model, we found that forced expression of BMP2 in HCT116 cells suppressed tumor growth, accompanied by decreased proliferative activity. Collectively, our results strongly suggest that BMP2 plays an inhibitory role in controlling the proliferation and aggressive features associated with colon cancer cells.
